# Commentary: Automated Machine Learning Model Development for Intracranial Aneurysm Treatment Outcome Prediction: A Feasibility Study

**DOI:** 10.3389/fneur.2022.878091

**Published:** 2022-06-10

**Authors:** Markus Huber, Markus M. Luedi, Lukas Andereggen

**Affiliations:** ^1^Department of Anaesthesiology and Pain Medicine, Inselspital, Bern University Hospital, University of Bern, Bern, Switzerland; ^2^Department of Neurosurgery, Kantonsspital Aarau, Aarau, Switzerland; ^3^Faculty of Medicine, University of Bern, Bern, Switzerland

**Keywords:** AutoML, stroke, machine learning, intracranial aneurysm, endovascular treatment

We read with great interest the article by *Ou and colleagues* (1) reporting on the application of an automated machine learning (AutoML) approach to predict recanalization after endovascular aneurysm occlusion. The authors are commended on accounting for key factors in outcome prediction such as (i) imbalanced datasets (2) by considering both the area under precision-recall curve (AUPRC) and the area under receiver-operating characteristic curve (AUROC), as well as the F1-score, (ii) the risk of overfitting by performing repeated cross-validations of the training and evaluation procedure, (iii) including graphical illustrations of the model building procedure as suggested in the literature (3) and (iv) providing code examples (4). Their results underlines the increased predictive performance of an AutoML approach compared to traditional logistic regression and a typical machine learning algorithm (Random Forest). Given the high predictive performance and the ease of using statistical software—as exemplified by the code and procedures in the Python language—the AutoML tool might provide a tool to bridge the implementation gap of such methods in medical practice (5).

From our own experience, we found the following points critical in applying ML models in outcome prediction.

While the discriminatory ability of the AutoML approach is highest among the statistical approaches in the study presented, the authors did not assess the calibration of the various algorithms. Calibration gives an estimate of how well the observed outcomes and predictions agree and are crucial in the clinical decision-making (6–8), thus we argue that an assessment of the calibration could be a further step to both evaluate and compare classical statistical methods with AutoML approaches to provide a more holistic estimate of the performance of various classifiers. As it is argued that one of the main advantages of AutoML is the possibility for non-ML experts to utilize ML models without prior know-how, we would like to point out that the application of AutoML as exemplified in the software code in Figure 4 of the paper still requires rather profound knowledge of the hyperparameters of the algorithm used in the model building pipeline—in the present application more than a dozen parameters need to be set. Thus, while the AutoML framework hides most of the parameter tuning and feature selection in a more easy-to-use software wrapper, a certain essential knowledge of ML—such as the concept of hyperparameters and cross-validation—is still required from the user to obtain robust and unbiased results. The authors mention further drawbacks of an AutoML approach, for example in terms of the black-box problems, which could be tackled by novel interpretation techniques such as SHAP values. However, while these techniques provide information regarding the importance of individual predictors, we argue that by considering the predictive performance of an ensemble of classifiers for two performance metrics jointly provides additional valuable information to compare different algorithms (9). Thus, an illustration of the performance of various algorithms within the search for the optimal pipeline of an AutoML application might provide additional and helpful information regarding the performance and robustness of both standard statistical methods such as multivariable logistic regression and modern machine learning methods.

From a clinical perspective, recanalization and recurrences following endovascular therapy of intracranial aneurysms is not infrequently encountered. The authors indeed list the number of patients analyzed and the short-term follow-up as a study limitation. However, the short follow-up time limits its validity. Although it has been shown that coiled aneurysms that showed complete occlusion at 6 months remained stable in most cases, up to 6.5% of those aneurysm occluded completely at 6-month later showed a recanalization (10). To evaluate recurrences rates dictating the treatment effectiveness after coiling, long-term follow-up is thus warranted (11). Although a low risk of rupture of coiled aneurysms with a follow-up period of up to 20 years have been described, larger aneurysms need to be followed for a longer time period (10, 12), as do aneurysms with residual filling after the initial treatment (13). Delayed recanalization, although rare, and the possibility of de novo aneurysm formation, however calls for continuous monitoring beyond 36 months (14).

We commend the authors on presenting an interesting and important application of a novel ML approach applicable for non-AI-experts that outperforms the commonly used statistical methods in predicting treatment outcome, as the latter is of utmost importance in any clinical practice evaluating its treatment outcomes.

## Author Contributions

MH, MML, and LA designed and wrote the initial draft. All authors contributed to the study design and critically revised the commentary. All authors contributed to the article and approved the submitted version.

## Funding

Open access funding was provided by the University of Bern.

## Conflict of Interest

The authors declare that the research was conducted in the absence of any commercial or financial relationships that could be construed as a potential conflict of interest.

## Publisher's Note

All claims expressed in this article are solely those of the authors and do not necessarily represent those of their affiliated organizations, or those of the publisher, the editors and the reviewers. Any product that may be evaluated in this article, or claim that may be made by its manufacturer, is not guaranteed or endorsed by the publisher.
