# Erratum: Commentary: Automated machine learning model development for intracranial aneurysm treatment outcome prediction: A feasibility study

**DOI:** 10.3389/fneur.2023.1183675

**Published:** 2023-03-21

**Authors:** Frontiers Production Office

**Affiliations:** Frontiers Media SA, Lausanne, Switzerland

**Keywords:** AutoML, stroke, machine learning, intracranial aneurysm, endovascular treatment

An omission to the funding section of the original article was made in error. The following sentence has been added: “Open access funding was provided by the University of Bern.”

The original article has been updated.

